# Evaluation of Antioxidant, Anti-Inflammatory and Cytoprotective Properties of Ethanolic Mint Extracts from Algeria on 7-Ketocholesterol-Treated Murine RAW 264.7 Macrophages

**DOI:** 10.3390/antiox7120184

**Published:** 2018-12-06

**Authors:** Fatiha Brahmi, Thomas Nury, Meryam Debbabi, Samia Hadj-Ahmed, Amira Zarrouk, Michel Prost, Khodir Madani, Lila Boulekbache-Makhlouf, Gérard Lizard

**Affiliations:** 1 Laboratoire de Biomathématique, Biochimie, Biophysique et Scientométrie, Faculté des Sciences de la Nature et de la Vie, Université de Bejaia, Bejaia 06000, Algerie; khodir.madani@univ-bejaia.dz (K.M.); lila.boulekbache@univ-bejaia.dz (L.B.-M.); 2 Laboratoire Bio-peroxIL ‘Biochimie du Peroxysome, Inflammation et Métabolisme Lipidique’ EA 7270/Inserm, Faculté des Sciences Gabriel, Université de Bourgogne Franche-Comté, 6 Bd Gabriel, 21000 Dijon, France; thomas.nury@u-bourgogne.fr (T.N.); debbabi.meryam55@gmail.com (M.D.); hjsamia@yahoo.fr (S.H.-A.); 3 Lab-NAFS ‘Nutrition—Alimentation Fonctionnelle & Santé Vasculaire’, Université Monastir, LR12ES05, Monastir 4000, Tunisie; 4 Laboratoire de Biochimie, Faculté de Médecine, Université de Sousse, Sousse 4000, Tunisie; zarroukamira@gmail.com; 5 Kirial International/Laboratoires Spiral, 21560 Couternon, France; michelprost.spiral@wanadoo.fr

**Keywords:** *Mentha* sp. ethanolic extracts, phenolic compounds, flavonoids, carotenoids, 7-ketocholesterol, antioxidant activity, anti-inflammatory activity, cytoprotection

## Abstract

The present study consisted in evaluating the antioxidant, anti-inflammatory and cytoprotective properties of ethanolic extracts from three mint species (*Mentha spicata* L. (MS), *Mentha pulegium* L. (MP) and *Mentha rotundifolia* (L.) Huds (MR)) with biochemical methods on murine RAW 264.7 macrophages (a transformed macrophage cell line isolated from ascites of BALB/c mice infected by the Abelson leukemia virus). The total phenolic, flavonoid and carotenoid contents were determined with spectrophotometric methods. The antioxidant activities were quantified with the Kit Radicaux Libres (KRL^TM^), the ferric reducing antioxidant power (FRAP) and the 2,2-diphenyl-1-picrylhydrazyl (DPPH) assays. The MS extract showed the highest total phenolic content, and the highest antioxidant capacity, while the MR extract showed the lowest total phenolic content and the lowest antioxidant capacity. The cytoprotective and anti-inflammatory activities of the extracts were quantified on murine RAW 264.7 macrophages treated with 7-ketocholesterol (7KC; 20 µg/mL: 50 µM) associated or not for 24 h and 48 h with ethanolic mint extracts used at different concentrations (25, 50, 100, 200 and 400 µg/mL). Under treatment with 7KC, an important inhibition of cell growth was revealed with the crystal violet test. This side effect was strongly attenuated in a dose dependent manner with the different ethanolic mint extracts, mainly at 48 h. The most important cytoprotective effect was observed with the MS extract. In addition, the effects of ethanolic mint extracts on cytokine secretion (Interleukin (IL)-6, IL-10, Monocyte Chemoattractant Protein (MCP)-1, Interferon (IFN)-ϒ, Tumor necrosis factor (TNF)-α) were determined at 24 h on lipopolysaccharide (LPS, 0.2 µg/mL)-, 7KC (20 µg/mL)- and (7KC + LPS)-treated RAW 264.7 cells. Complex effects of mint extracts were observed on cytokine secretion. However, comparatively to LPS-treated cells, all the extracts strongly reduce IL-6 secretion and two of them (MP and MR) also decrease MCP-1 and TNF-α secretion. However, no anti-inflammatory effects were observed on 7KC- and (7KC + LPS)-treated cells. Altogether, these data bring new evidences on the potential benefits (especially antioxidant and cytoprotective properties) of Algerian mint on human health.

## 1. Introduction

Cholesterol oxide products (also named oxysterols) are oxidized forms of cholesterol which can be formed by auto-oxidation, enzymatically or both [1,2]. A number of these products, mainly those oxidized at C7, such as 7-ketocholesterol (7KC), which is mainly formed by auto-oxidation, are biologically active molecules and can have various side effects at elevated concentrations [3,4]. 7KC is known to induce a complex mode of cell death, named oxiapoptophagy [5,6], which is characterized by an overproduction of reactive oxygen species (ROS), an induction of apoptosis and autophagy [5,7]. In addition, 7KC induces inflammatory processes in various cell types including monocytes [8]. 7KC is thought to contribute to the development of various major age-related diseases: atherosclerosis and cardiovascular diseases, Alzheimer’s disease (including vascular dementia), age-related macular degeneration and cataract, and certain forms of cancers (colon carcinoma, breast cancer) [9,10,11,12]. 

On the other hand, numerous chronic inflammatory immune diseases are mediated by 7KC through the increased generation of bioactive mediators such as cytokines and free radicals from immune cells or tissue cells under the action of various environmental factors, which can be of physical, chemical or biological origins [13,14,15]. Furthermore, monocytes/macrophages (and microglial cells, which are the equivalent of monocytes/macrophages in the brain), are activated in the presence of 7KC with an induction of oxidative stress leading to hydrogen peroxide (H_2_O_2_) and superoxide anion (O_2_^•−^) overproduction, lipid peroxidation, plasma membrane alteration, organelles dysfunctions (mitochondria, peroxisome and lysosome), DNA damage (8-oxoguanine formation, internucleosomal DNA fragmentation) and cytokine secretion (mainly IL-8 secretion) leading to cell death [16,17,18]. On the other hand, 7KC is increased in the gut wall in inflammatory-related diseases [19] and is known to trigger pro-inflammatory cytokine secretion and to enhance adhesion molecules expression on monocytic and endothelial cells [20,21]. 

To counter the deleterious effects of 7KC including oxidative stress, inflammation and cell death induction, it is therefore necessary to identify cytoprotective compounds which could be further used to develop pharmacological strategies in pathologies associated with increased 7KC levels [22]. At the moment, various plant-derived bioactive molecules are thought to reduce the inflammatory response of activated macrophages [14]. Phenolic compounds have strong in vitro and in vivo antioxidant activities associated with their ability to scavenge free radicals, break radical chain reactions and chelate metals [23]. Carotenoids could also have protective effects against degenerative or cardiovascular diseases, and are known for their antioxidant activity [24].

Worldwide and in Algeria, many plants have been eaten since ancient times, and some have medicinal properties as well as nutritional values. It is believed that the consumption of these plants can cure illness and diseases. Among these plants, mints (*Mentha* sp.) have been among the most widely used aromatic plants for food flavoring, tea preparation and as medicines since ancient times [25]. Mint exerts a wide range of biochemical and pharmacological effects, most of which are thought to be due to the presence of a wide range of well-defined phytochemicals that exhibit anti-inflammatory [26], anti-carcinogenic [27], antioxidant and anti-peroxidant properties [28,29]. Among these phytochemicals, phenolic compounds and carotenoids include many different families of aromatic secondary metabolites in plants. Interestingly, mint extracts at their bioactive dilutions had no observable toxicity in our previous study [30]. 

In the present study, we determined the phenolic, flavonoid, and carotenoid contents of three ethanolic mint extracts (*M. spicata*, *M. pulegium* and *M. rotundifolia* extracts) with spectrophotometric methods. The antioxidant activity of these extracts were also measured using three complementary assays: the KRL^TM^ (Kit Radicaux Libres), the FRAP (Ferric Reducing Antioxidant Power) and the DPPH (2,2-diphenyl-1-picrylhydrazyl) assays. As we established that mint extracts have antioxidant properties, and as it is known that 7KC-induced cell death is associated with oxidative stress leading to cell death, we evaluated the ability of mint extracts to counteract 7KC-induced cell death. In addition, as 7KC is known to promote inflammation associated with an induction of cytokine synthesis [31], the impact of mint extracts on cytokine secretion (IL-6, IL-10, MCP-1, IFN-γ, TNF-α) was also measured. To this end, RAW 264.7 murine macrophage cells were cultured in the absence or presence of 7KC associated or not with mint extracts to evaluate the ability of these extracts to prevent cell death and inflammatory cytokine secretion. The anti-inflammatory effects of mint extracts were also evaluated on lipopolysaccharide (LPS) and (7KC + LPS)-treated RAW 264.7 cells, since LPS (a major compound of gram negative cells) can affect the metabolism of macrophages which is a main target of 7KC [8,32].

## 2. Materials and Methods 

### 2.1. Herbal Material 

The leaves and herbarium samples of *Mentha spicata* (BR 0000006946043), *Mentha pulegium* (BR 0000006946043) and *Mentha rotundifolia* (BR 0000006946197) were collected from the Smaoun region (36°37′0″ N, 4°48′0″ E) of Bejaia, Algeria in June 2013. The samples were previously authenticated by Professor J. Lejoly in the Laboratory of Systematical Botany and Phytosociology, Free University of Brussels (ULB), Belgium. Voucher specimens were deposited in the Herbarium of the National Botanical Garden of Meise (Brussels, Belgium).

### 2.2. Preparation of Ethanolic Mint Extracts

The leaves were air dried at room temperature to obtain constant weights. The dried material was then comminuted into fine powder using the waring commercial laboratory blender (IKA^®^, Staufen, Germany). A known quantity of the powder (20 g) was extracted by stirring with 1000 mL of ethanol/water (1:1, *v*/*v*), at room temperature and at 130 rpm for 24 h. The crude extracts were filtered through Watman No. 1 paper, concentrated with a rotary vacuum evaporator (40 °C), lyophilized and maintained in the dark at 4 °C until tested. The extraction yield was calculated as follows: Yield (%) = W_1_/W_2_ × 100, where W_1_ was the weight of extract after lyophilization and W_2_ was the weight of the dried powder of plant material. The final amount of dry aqueous ethanol extracts of *M. spicata* (MS), *M. pulegium* (MP) and *M. rotundifolia* (MR) were 0.11 g (27.3%), 0.09 g (24.3%), and 0.12 g (29.5%), respectively (Table 1).

### 2.3. Quantification of Phenolic, Flavonoid and Carotenoid Contents of Ethanolic Mint Extracts

The total phenolics content of the extracts was determined spectrophotometrically according to the Folin–Ciocalteu colorimetric method as described in our previous study [28]. One hundred μL of extract were transferred to a 10.0 mL volumetric flask containing 6.0 mL of water, to which were subsequently added 500 μL of undiluted Folin–Ciocalteu reagent; after 1 min, 1.5 mL of 20% aqueous Na_2_CO_3_ were added, and the flask was brought to volume with water. After 2 h incubation at 25 °C, the absorbance was measured at 760 nm versus a blank prepared without extract. The total phenolics content was expressed as mg gallic acid equivalents (GAE)/g dry weight.

The flavonoid content in the extracts was determined spectrophotometrically using the method based on the formation of a complex flavonoid-aluminum as reported in our previous study [28]. An amount of 1.5 mL of a 2% AlCl_3_ solution was added to 1.5 mL of extract. After 15 min at RT in the dark, the absorbance was measured at 430 nm versus a blank prepared without extract. The total flavonoids content was calculated as a quercetin (mg/g) equivalent. 

Carotenoids from ground mints were extracted by procedures described previously [33]. A quantity of plants powder (1 g) was added to 20 mL of solvent mixture (hexane, acetone and ethanol, 1:2:1, *v*/*v*/*v*). After 40 min of stirring, the absorbance of upper phase was determined at 450 nm by UV-Vis spectrophotometer (Shimadzu, Marne la Vallée, France). β-Carotene was used as the standard and the results were expressed as mg β-carotene equivalent per g dry weight of plant (βCE/g DW), through the β-carotene calibration curve.

### 2.4. Determination of the Antioxidant Capacity of Ethanolic Mint Extracts

The antioxidant activity of ethanolic extracts obtained from *Mentha* was evaluated using the KRL^TM^ biological test (Kit Radicaux Libres, Kirial International/Laboratoires Spiral, Couternon, France) [34,35,36], and the chemical techniques that include the ferric reducing antioxidant power (FRAP) and the 2,2-diphenyl-1-picrylhydrazyl (DPPH) assays. The protocols used have been previously described by Debbabi et al. [22]. 

### 2.5. Cells and Cell Treatments 

RAW 264.7 cells were seeded at 240,000 cells per well in 24-well microplates containing 0.5 mL of culture medium (Dulbecco’s modified Eagle medium (DMEM); Lonza, Basel, Switzerland) supplemented with 5% (*v*/*v*) heat-inactivated fetal bovine serum (FBS) (Pan Biotech, Aidenbach, Germany) and 1% antibiotics (100 U/mL, penicillin, 100 mg/mL streptomycin) (Pan Biotech). Cells were incubated at 37 °C in a humidified atmosphere containing 5% CO_2_, and passaged twice a week. 

In the present study, RAW 264.7 cells were previously cultured for 24 h. They were further cultured for 24 h and 48 h in the presence of 7KC (20 µg/mL; 50 µM) without or with mint extracts. Mint extracts and 7KC (Supplementary Figure S1) were simultaneously added.

### 2.6. Evaluation of Cell Growth: Staining with Crystal Violet

Adherent cells were quantified by staining with crystal violet [6]. RAW 264.7 cells were seeded in 24-well plates, and cultured without or with mint extracts (25, 50, 100, 200 and/or 400 µg/mL) combined with 7-KC (20 µg/mL) for 24 h and 48 h. The assays were realized twice in triplicate.

### 2.7. Multiplexed Flow Cytometric Analysis of Inflammatory Cytokines

The production of inflammatory cytokines was investigated in the culture medium of RAW 264.7 cells, which were either untreated or treated for 24 h with different concentrations of mint extracts (200 and 400 µg/mL) associated or not with 7KC (20 µg/mL; 50 µM). Cells only treated with 7KC (20 µg/mL) were also used. The ability of these extracts to induce cytokine secretion (MCP-1, IL-6, IL-10, TNF-α, and IFN-γ) was investigated by a flow cytometric bead-based assay, i.e., the multiplexed Cytometric Bead Array (CBA) (BD-Biosciences, San Jose, CA, USA) method as previously described [31]. Cytokine values lower than the limit of detection provided by BD-Biosciences (IL-6: 5 pg/mL; IL-10: 17.5 pg/mL; MCP-1: 52.7 pg/mL; IFN-γ: 2.5 pg/mL; TNFα: 7.3 pg/mL) were noted as not detectable (ND).

### 2.8. Statistical Analysis 

Crystal violet values were compared with a Mann—Whitney test. Cytokine levels obtained by the CBA were analyzed by using a one-way analysis of variance (ANOVA) followed by a *t*-test [31]. *p* < 0.05 were considered as statistically significant.

## 3. Results

### 3.1. Quantification of Antioxidants and Determination of the Antioxidant Capacity 

Among the three extracts, *M. spicata* (MS) extract contained the highest (30.8 ± 3.0 mg GAE (Gallic Acid Equivalent)/g DM (Dry Matter)) amount of total phenolic compounds (TPC). The concentrations of carotenoids and flavonoids were highest in the *M. rotundifolia* (MR) extract. However, this extract showed the lowest concentration of total phenolic compounds with a significant difference (*p* < 0.05) (Table 1). 

Several methods were used to evaluate the antioxidant activities of mint extracts. Up to now, the KRL^TM^ assay, which is a biological test based on the lysis of red blood cells, has never been used on mint extracts. The data obtained in the present study demonstrate that the three mint species possess antioxidant activities. With the KRL^TM^ test, the data obtained support that the extracts have the ability to protect the plasma membrane of red blood cells against oxidative stress. The compounds present in MS and MR extracts are more able to reduce the damages caused by free radical attack on erythrocytes than MP extract. 

The ferric reducing power of the mint extracts, determined with the FRAP assay, was found to be dose-dependent. MS extract, which contained the highest amount of total phenolic compounds, was the most potent reducing agent, whereas MR extract, which contained the lowest amount, showed the weakest activity. The third antioxidant assay used was DPPH, which is often employed to estimate the free radical scavenging activity of plant extracts. With the DPPH assay, MS extract showed the best scavenging capacity of DPPH^•^ radical followed by MP and MR extracts. In addition, in this study, the activity of the MS extract shown with the FRAP and DPPH assays exceeded that of Trolox^®^ (Sigma-Aldrich, St Quentin Fallavier, France) used as standard (Table 2).

We noticed that the determined values of the antioxidant activity using the three different assays varied widely. Several studies have showed that the antioxidant activity assessed relies highly on the test system employed. The antiradical activity determined by the DPPH test characterizes the ability of compounds to react with free radicals, giving information on the radical scavenging or antiradical activity, although in many cases this activity does not correspond to the antioxidant activity. In spite of the wide use of DPPH, this test system in some cases gives incorrect results. It is necessary to note that, in the DPPH test system, hydrophobic antioxidants show low reactivity. On the contrary, the antioxidant activity evaluated by the Ferric ions (Fe^3+^) reducing antioxidant power (Fe^3+^-Fe^2+^ transformation assay) method represents the ability to inhibit the process of oxidation [37].

The KRL^TM^ test gives an indication on the true antioxidant activity with regard to lipids or food stabilization, the conduction of a study on the biological simple allows the evaluation of red blood cell resistance against the free radicals induced by 2,2′-azobis (2-amidinopropane) hydrochloride (AAPH) that acts by producing peroxyl radicals, which induce lipid and protein peroxidation in the cell membrane. All antioxidants in the blood are mobilized to fight off the radical attack, and, therefore, this assay provides an overall appraisal of the antioxidant status [38]. Therefore, in the KRL test, the MR extract with the lowest total phenolic content was more effective than MP in protecting erythrocytes. This can be explained by not only the nature of the method used (biological test) but also by the quality of the compounds present in the extracts and not by their contents. Indeed, MR extract was characterized by its richness in flavonoids. 

### 3.2. Effect of Mint Extracts on 7-Ketocholesterol-Induced Cell Growth Inhibition

**Figure 1:**
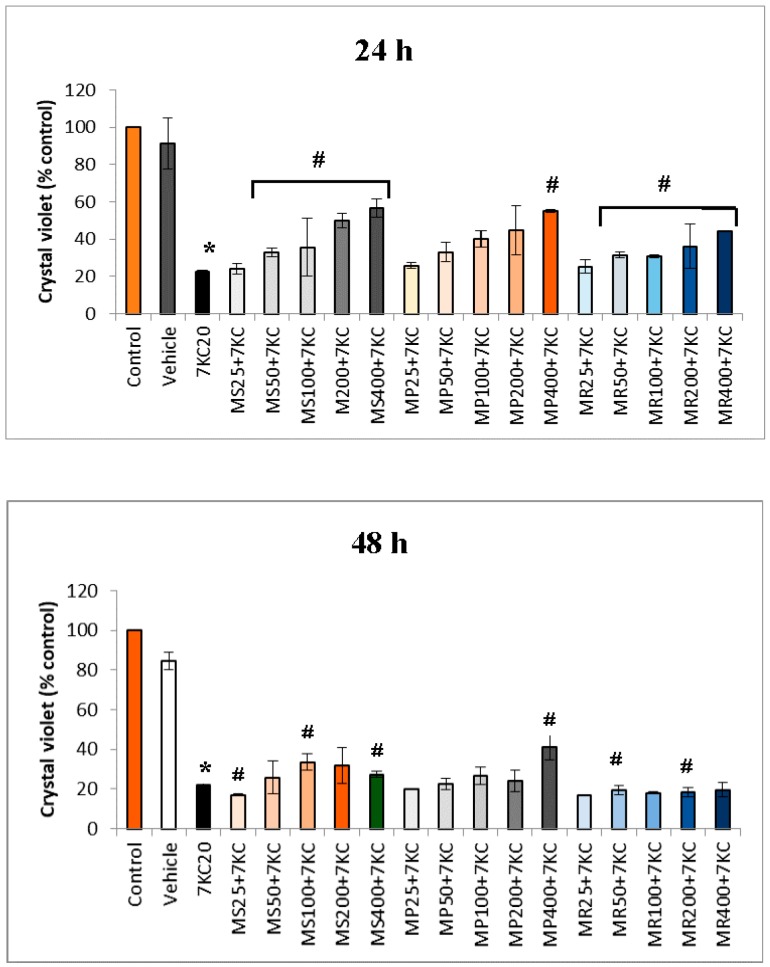
Attenuation with ethanolic mint extracts of 7-ketocholesterol (7KC)-induced cell growth inhibition. The effects of *Mentha* extracts were evaluated on 7KC (20 µg/mL (50 µM); 24 h and 48 h)-treated RAW 264.7 cells with the crystal violet test, which allows the quantification of adherent cells. After 24 h of culture, mint extracts and 7KC were simultaneously added to the culture medium. Ethanol 0.1% (used as vehicle) corresponds to the highest ethanol concentration used to dissolve 7KC. Data are mean ± Standard Deviation (SD) from two independent experiments conducted in triplicate. * Significant difference between vehicle and 7KC (Mann Whitney; *p* < 0.05). **#** Significant difference between 7KC and (7KC+ethanolic mint extract) (Mann Whitney; *p* < 0.05).

RAW 264.7 cells, previously cultured for 24 h, were further cultured for 24 h and 48 h in the presence of 7KC (20 µg/mL; 50 µM) without or with mint extracts (Figure 1). Mint extracts and 7KC were simultaneously added in the culture medium. The impact of 7KC on cell growth was evaluated with the crystal violet test. At the concentration used, 7KC significantly inhibits cell growth both at 24 and 48 h. The data indicate that mint extracts were effective in protecting RAW 264.7 cells from 7KC-induced cytotoxicity in a dose dependent manner especially at 24 h. At 48 h, slight cytoprotective effects were observed, especially with MS and MP extracts. 

### 3.3. Effect of Mint Extracts on Cytokine Secretion Induced by 7-Ketocholesterol (7KC) and Lipopolysaccharide (LPS) Associated or Not with 7KC

7KC and LPS are potent inducers of inflammation [31,39]. In the present study, we determined the effects of mint extracts on cytokines secretion on murine macrophages RAW 264.7 when they were untreated or treated for 24 h with 7KC (20 µg/mL), LPS (0.2 µg/mL) and LPS associated or not with 7KC. 

**Figure 2:**
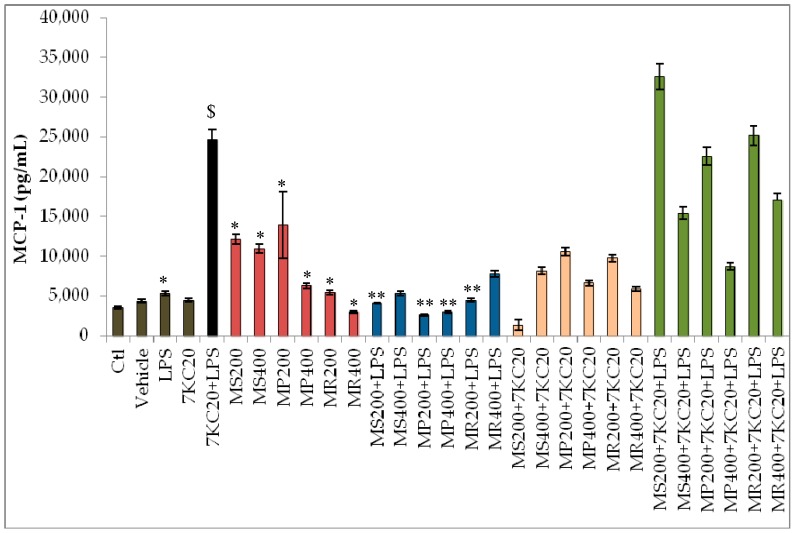

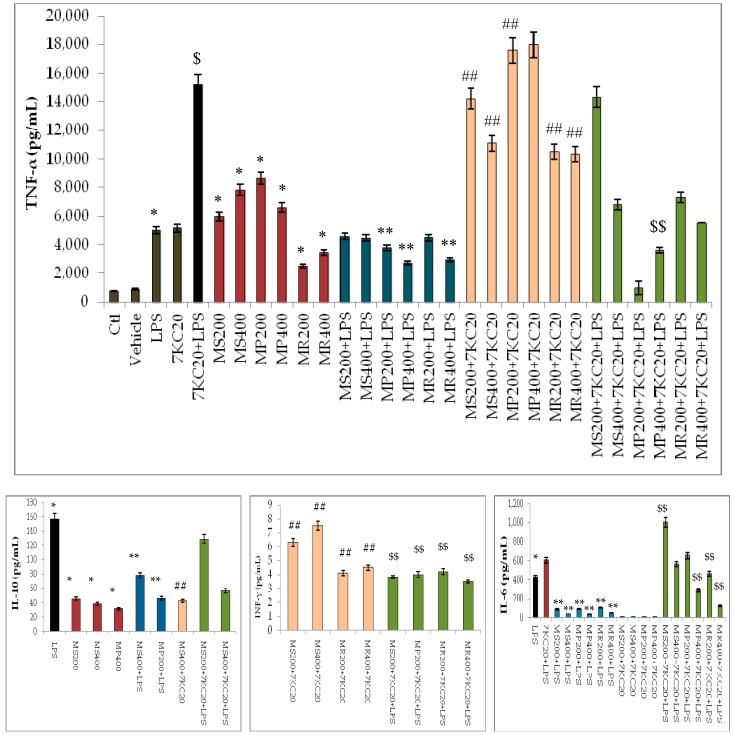
Effect of mint extracts on cytokine secretion. Data shown are mean ± SD from three independent experiments. They were analyzed by the ANOVA’s test followed by a t test. A *p*-value of 0.05 or less was considered as statistically significant (*p* < 0.05). No significant differences were observed between control (Ctl; untreated cells) and vehicle (ethanol 0.1%). *: comparison lipopolysaccharide (LPS), *Mentha spicata* (MS), *Mentha pulegium* (MP) and *Mentha rotundifolia* (MR) LPS, MS, MP and MR extracts versus Ctl; #: comparison 7KC20 versus vehicle; $: comparison (7KC20 + LPS) versus vehicle; **: comparison (MS, MP or MR extracts + LPS) versus LPS; ##: comparison (MS, MP or MR extracts + 7KC20) versus 7KC20; $$: comparison (MS, MP or MR extracts + 7KC20 + LPS) versus (7KC20 + LPS). Cytokine values lower than the limit of detection (IL-6: 5 pg/mL; IL-10: 17.5 pg/mL; MCP-1: 52.7 pg/mL; IFN-γ: 2.5 pg/mL; TNFα: 7.3 pg/mL) were considered as not detectable, and are not shown (for details see supplementary Table S1). MCP-1 and TNF-α were detected at high levels in all conditions of treatments. However, IL-6, IL-10 and TNF-α secretions were detected at lower levels, and were often not detectable in several conditions; in those conditions, the missing values in the abscissa correspond to non-detectable values (for details, see supplementary Table S1).

The plant extracts showed a complex activity on cytokine secretion which depends on the cytokine considered and whether the cells are only treated with mint extracts or simultaneously treated with mint extracts associated either with LPS or with (7KC + LPS) (Figure 2). Due to the important variations in cytokines secretion, the details of the values, which are presented in the Figure 2, are shown in supplementary material (Supplementary Table S1).

In untreated RAW 264.7 cells and in vehicle-treated cells, similar constitutive secretions of MCP-1 and TNF-α were observed; no IL-6, IL-10 or IFN-ϒ secretion were detected (Figure 2). The constitutive secretion of MCP-1 and TNF-α is in agreement with previous data [40]. In the presence of LPS, the secretion of MCP-1 and TNF-α was strongly enhanced, and an induction of IL-6 and IL-10 secretion was detected (Figure 2); the cytokine levels are in the range of those previously described on LPS-treated RAW 264.7 cells [40,41]. 

In addition, as reflected in Figure 2, we clearly observed that MCP-1 and TNF-α values increased upon treatment with the three mint extracts compared to control and vehicle. Hence, we hypothesize that substances present in these extract might be contributing to this cytokine release property in murine macrophages (RAW 264.7).

Consequently, it is considered that mint extracts could stimulate the healthy immune system by increasing inflammatory cytokine production. Thus, these extracts could be valuable for the activation of the immune system and in the inflammatory procedure in healthy persons or in patients with different illnesses. Further studies are needed on other cell types for a better understanding of the molecular mechanisms of action of mint extracts towards stimulation of secretion of these pro-inflammatory cytokines.

In addition, we observed that the effects of the investigated mint extracts are different regarding the pro-inflammatory cytokine secretion (MCP-1, TNF-α, IL-6 and INF-γ). At the moment, no rational explanation is available and additional studies are required to explain this complex effect. On the other hand, we found that the level of the anti-inflammatory cytokine IL-10 was not increased. It is obvious that the most marked effects were observed on the most expressed cytokines (MCP1 and TNF-α). The other cytokines present in smaller quantities are modulated, but the effect was more difficult to observe; this may be due to the model used.

The most pronounced inflammatory profile was observed in the presence of (7KC + LPS): IL-6, MCP-1 and TNF-α were significantly increased comparatively to untreated and vehicle-treated cells, and the level of these cytokines was also higher than when the cells were only treated with LPS or with 7KC (Figure 2). Comparatively to untreated cells, the three mint extracts induce an increased secretion of MCP-1 and TNF-α (Figure 2). However, in the presence of LPS, the mint extracts attenuate cytokine secretion. Thus, the three mint extracts decrease the secretion of IL-6 which can be associated with decreased levels of MCP-1 and TNF-α (Figure 2). However, in the presence of 7KC and (7KC + LPS), mint extracts rather contribute to enhance cytokines secretion (Figure 2). Thus, mint extracts have both immunostimulatory effects (increased secretion of MCP-1 and TNF-α) and anti-inflammatory activities towards LPS. In addition, our data support that the anti-inflammatory activity of mint extracts could depend on the pro-inflammatory agent considered. 

## 4. Discussion

The plants studied are characterized by their richness in antioxidants (phenolics and carotenoids). It is difficult to compare the total phenolic contents (TPC) found in this study with those in our previous study [28], in which we removed interfering compounds (e.g., carotenoids, chlorophylls, reducing sugars or ascorbic acid), thus leading to an overestimation of the TPC. 

This pretreatment facilitated the identification of the nature of flavonoids and phenolic acids and their quantification in these plants by high performance liquid chromatography with diode array detection (HPLC/DAD)in our previous work [28]. 

Antioxidant contents are generally related to the antioxidant activity. Therefore, the extracts have significant antioxidant power. Since secondary metabolites and antioxidant activity are involved in the effect on the 7 KC, we have found it useful to test the impact of the extracts of these plants on this oxysterol. It is important to emphasize that the use of 7KC (25–50 µM) on different cell types is a relevant model: (a) to evaluate the relationship between oxidative stress, apoptosis and autophagy; (b) to specify the part played by the organelles (mitochondria, peroxisomes and lysosomes) in these processes; (c) to determine the interactions between these organelles, and (d) to identify natural and synthetic molecules able to prevent 7KC-induced side effects [22]. In addition, mint extracts were used in a range of non-toxic concentrations: (25 to 400 µg/mL) to study their effects on cell death, and (200 and 400 µg/mL) to determine their impact on cytokine secretion [30].

Altogether, the present study establishes that the extracts of *M. spicata* (MS), *M. pulegium* (MP) and *M. rotundifolia* (MR) have antioxidant and cytoprotective effects when used on murine macrophages RAW 264.7 treated with 7KC, which is known to trigger oxidative stress and cell death [3,8,13].

As previously reported, the mint extracts studied contain a considerable amount of antioxidants (phenolics and carotenoids) [28]. The biological activity of polyphenols and carotenoids in the different systems is believed to be due to their RedOx properties, which includes the absorption and neutralization of free radicals [24,42]. Therefore, the antioxidant effects of the three mint extracts used were compared with those of Trolox^®^ used as positive reference in the KRL^TM^, FRAP and DPPH assays. The antioxidant capacity of mint extracts might be due to their hydrogen-donating ability [29]. The results obtained in this study were consistent with our previous results [28]. However, the scavenging capacity is even stronger because of the presence of compounds other than phenolic compounds, such as carotenoids, which are also powerful antioxidants and which were eliminated in our previous study. In addition, our results are in line with other studies. For example, among all of the extracts analyzed by Conforti et al. [43], *Mentha* extracts are the richest in total phenolics and showed the best radical scavenging and antioxidant activities. Carotene and other carotenoids, such as lycopene or the xanthophylls, also exert antioxidant functions, such as quenching of singlet oxygen and other electronically excited molecules that are produced by photoexcitation or chemiexcitation reactions. They further react with peroxyl or alkoxyl radicals [44,45], and are reported to reduce disease-associated chronic health problems [46].

7KC, which is mainly obtained by cholesterol autoxidation, is also present in various food constituents and can be formed endogenously [6,17,47]. Currently, the ability of 7KC to induce apoptosis is well established, and the involvement of oxidative stress in 7KC-induced cell death is supported by numerous arguments [48,49] such as the ability of α-tocopherol to counteract oxiapoptophagy, the particular mode of cell death induced by 7KC on 158N murine oligodendrocytes [7]. As it is known that several fruits and vegetables contain high amounts of antioxidants that may promote human health or lower the risk of age related-diseases associated with increased levels of 7KC [17], it was of interest to evaluate the impact of mint extracts on 7KC-induced side effects. In this respect, *Mentha* sp is among the most promising plants for use against several diseases in which the pathogenesis is at least partly attributed to ROS [26,27,50]. Previous studies have shown that mint extracts were able to prevent the propagation of lipid peroxidation in a complex lipid matrix, such as a foodstuff or biological membrane. In his study, Janoszka [51] showed that the intensity of the influence of vegetables and spices on the formation of oxysterols depended both on the type and concentration of antioxidants. It is supposed that the cytoprotective effect of mint extracts may be connected with the presence of antioxidants. In agreement with our previous studies, MS has the most potent antioxidant properties and it is also very efficient to prevent the cytotoxic effect of 7KC on RAW 264.7 cells as shown with the crystal violet test. Its activity could be relied on with the high amount of total phenolic acids, and especially rosmarinic acid, in the extract of this plant [28,52]. Indeed, various herbs or vegetable extracts have been reported as antioxidants, thanks to their polyphenolic content, in edible oils and meat products [51]. The study of Chien et al. [53], who investigated heat-induced cholesterol oxidation by incorporating quercetin, showed that adding this flavonoid to cholesterol was effective in inhibiting the formation of cholesterol oxidation products, including 7-KC. Due to their lipophilicity and to their specific ability to scavenge peroxyl radicals, carotenoids have also been implicated in the protection of cellular membranes and lipoproteins against oxidative damages. In parallel, several studies have investigated the impact of supplementation with these dietary compounds on cardiovascular diseases, given their free-radicals scavenger properties and their ability to improve the resistance of low-density lipoprotein cholesterol to oxidation [45]. Thus, the compounds present in mint extracts can scavenge the active forms of oxygen involved in the initiation steps of lipid oxidation or break the oxidative chain reaction. They are also able to react with fatty acid peroxyl radicals to form stable radicals, which are either insufficiently reactive for further reactions or to form non-radical products. Since cholesterol oxidation proceeds via a free radical mechanism similar to the oxidation of polyunsaturated fatty acids, antioxidants may also be able to prevent or retard the generation of cholesterol oxidation products [51]. 

Another property of the mint extracts studied on RAW 264.7 cells is their ability to modulate cytokine secretion. It has been supposed that mint extracts could have anti-inflammatory activities. Indeed, previous results obtained by Arumugam et al. [26] suggest that the ethanol fractions from the leaves of *M. spicata* attenuate chronic inflammation. Currently, the influence of mint extracts on the production of cytokines has not yet been reported but several natural products, which are present in mint, were tested for their pro- or anti-inflammatory activities by determining their impact on the cytokines secretion [54]. Mueller et al. [54] revealed that various plant extracts reduced IL-10 secretion, namely anise, apple, bay leaves, black pepper, cinnamon, ginger, holy basil, licorice, nutmeg, rooibos tea, rosemary, sage and thyme. Notably, several compounds were more efficient in the reduction of IL-6 and TNF-α secretion compared to cortisol (apigenin, capsaicin, chrysin, diosmetin, kämpferol, luteolin, myricetin, naringenin and quercetin); these compounds, however, did not simultaneously increase IL-10 secretion. In our study, under treatment with the three mint extracts at the concentrations of 200 µg/mL associated with 7-KC+ LPS, the IL-6 and MCP-1 levels are higher than with 7-KC + LPS. In those conditions, it appears that the mint extracts stimulate the immune system by enhancing the secretion of some cytokines through as yet unknown mechanisms. Further studies are needed to clarify how this activation happens. On the contrary, the extracts of the three mint species showed anti-inflammatory activities mainly when associated with LPS. Furthermore, we noticed that 7KC did not induce secretion of MCP-1, nor did its combination with mint extracts of MS and MP compared to the effect of extracts alone. A slight increase is found with the combination of 7KC only with the MR extract used at 200 and 400 μg/mL. 7-KC alone did not cause secretion of MCP-1 and these data are consistent with the results obtained on other cell types. Indeed, according to Dugas et al. [55], 7KC at 20 µg/mL enhances the secretion of IL-8 and reduces those of MCP-1; at 30 µg/mL, 7KC reduced the secretion of Vascular Endothelial Growth Factor (VEGF) and had no effect on IL-8 and MCP-1 secretion. The nature and the diversity of the substances present in the mint extracts could explain, at least in part, the complex effects on the different results found: some compounds are probably pro-inflammatory, whereas some others are anti-inflammatory or have no effects. The resulting effect reflects the complexity of the activity of mint extracts on cytokine secretion and the difficulty to determine whether ethanolic mint extracts are either pro-inflammatory or anti-inflammatory. The data obtained rather supposed that the impact of mint extracts on cytokine secretion could be influenced by environmental conditions. It should be also noted that phenolic compounds stimulated RAW 264.7 cells for marked release of biologically active TNF-α, being consistently well above positive control levels. This activity could depend on the structure of the phenolic compounds. Thus, shikimic acid stimulated RAW 264.7 cells only moderately for TNF-α release, but introduction of galloyl groups remarkably enhanced the amount of cytokine released [56]. Mint extracts have been shown to contain large amounts of polyphenolic compounds (flavonoids, phenolic acids and others), including rosmarinic acid [28,29]. In our previous study, the amounts of this phenolic acid quantified by HPTLC were: 3.63 ± 0.1% (*w*/*w*) for MS extract; 1.04 ± 0.05% for MP and 0.96 ± 0.03% for MR [52]. Rosmarinic acid have been shown to exert anti-allergic and anti-inflammatory effects by reducing the levels of inflammatory cytokines, chemokines, and anti-allergen antibodies [57]. In our previous study, rosmarinic acid was the major compound detected in all mint extracts [28,52]. According to Sanbongi et al. [58], this phenolic acid inhibits allergic inflammation induced by mite allergen in a mouse model; rosmarinic acid also inhibited the increased expression of IL-4 and IL-5, as well as the local expression of Th2 cytokines and chemokines and eotaxin in the lungs of sensitized mice. On the other hand, regarding carotenoids, there is a lot of data supporting their anti-inflammatory action. Ciccone et al. [45] reported that the antioxidant activity of *β*-carotene and lycopene prevent inflammatory oxidative stress and increased the biodisponibility of vascular nitric oxide. In addition, some authors have provided encouraging results in early atherosclerotic patients, in which serum carotenoids were inversely associated with inflammatory cytokines. Hence, by improving the lipid profile, suppressing lipid peroxidation and reinforcing the activity of the antioxidant system, the carotenoids are able to counter vascular wall inflammation and to stabilize membrane properties, thus reducing the risk of cardiovascular diseases. However, when mint extracts were associated with 7KC and (7KC + LPS), no reduction of cytokine secretion was observed. In addition, the ability of mint extracts to stimulate the production of MCP-1 and TNF-α has both a negative aspect, when we consider the impact on inflammatory diseases, and a beneficial aspect if we take into account the potential effect on the prevention of tumor development. Altogether, our data establish that mint extracts have both immunostimulatory effects (stimulation of the secretion of MCP-1 and TNF-α) and anti-inflammatory activities towards LPS that could be valued in phytotherapy. They also highlight that the ability of mint extracts to prevent or not cytokine secretion could depend on the inducer of inflammation considered, and could only be efficient on particular signaling pathways leading to inflammation.

## 5. Conclusions

This study underlines that the Algerian flora including the three *Mentha* species studied (*M. spicata*, *M. pulegium*, *M. rotundifolia*) can be an interesting source of compounds with antioxidant, cytoprotective and immunomodulatory properties. The compounds present in the ethanolic mint extracts (mainly polyphenols and carotenoids) have potential uses in different fields (food, cosmetics and pharmaceuticals), and could be of interest to prevent age-related diseases often associated with oxidative stress and inflammation. The effect of mint extracts was underlined in this study by their ability to protect murine RAW 264.7 macrophages from 7KC-induced toxicity, which is increased in major human diseases [9,10,13], to reduce LPS-induced cytokine secretion, especially IL-6 and TNF-α, and to stimulate the secretion of MCP-1 and TNF-α. Thus, our data provides new evidence on the potential health benefits of mint, which is frequently used as a culinary herb in North Africa, in the Middle East and also in the Mediterranean diet. The data obtained also extend our knowledge on the biological activities of mint extracts and on the possibility to use these extracts in phytotherapy.

## Figures and Tables

**Table 1:** Ethanol 50% extraction yields, total phenolic contents (TPC), flavonoid contents (TFC) and total carotenoid contents (TCC).

Extracts	Extraction Yield (%)	TPC ^(^*^)^ (Eq. mg Gallic Acid/g)	TFC ^(^*^)^ (Eq. mg Quercetin/g)	TCC ^(^*^)^ (Eq. mg β-Carotene/g)
*MS*	27.3	30.8 ± 3.0 ^a^	5.2 ± 0.4 ^b^	3.4 ± 0.1 ^b^
*MP*	24.3	28.3 ± 1.5 ^a^	5.7 ± 0.2 ^b^	3.3 ± 0.2 ^b^
*MR*	29.5	23. 8 ± 3.3 ^b^	7.1 ± 0.3 ^a^	4.2 ± 0.2 ^a^

^(^*^)^ Data from three independent experiments; all contents are expressed versus dry plant material. Values in the same column sharing different letters are significantly different (*p* < 0.05). *Mentha spicata* L. (MS), *Mentha pulegium* L. (MP) and *Mentha rotundifolia* (L.) Huds (MR). Eq: Equivalent.

**Table 2:** Antioxidant activity of ethanolic mint extracts using the Kit Radicaux Libres (KRL^TM^), the ferric reducing antioxidant power (FRAP) and the 2,2-diphenyl-1-picrylhydrazyl (DPPH) assays.

Antioxidant Assays	Antioxidant Activity (mg of Trolox equivalent/g)		
	MS	MP	MR
KRL	796.78 ± 2.72 ^a^	554.19 ± 2.46 ^c^	606.34 ± 1.09 ^b^
FRAP	1430.13 ± 221.66 ^a^	776.48 ± 39.48 ^b^	364.79 ± 32.52 ^c^
DPPH	207.96 ± 10.98 ^a^	81.98 ± 6.44 ^b^	38.50 ± 1.96 ^c^

Data are presented in Trolox Equivalent (TE). Data shown are mean of three independent experiments realized in triplicate. *Mentha spicata* L. (MS), *Mentha pulegium* L. (MP) and *Mentha rotundifolia* (L.) Huds (MR). Values in the same line sharing different letters are significantly different (Mann Whitney, *p* < 0.05).

## Supplementary Materials

The following are available online at http://www.mdpi.com/2076-3921/7/12/184/s1, Figure S1: 7-ketocholesterol (7KC) structure, Table S1: Effect of mint extracts on cytokine secretion.
