# Supplementary material for: Evaluation of Antioxidant, Anti-Inflammatory and Cytoprotective Properties of Ethanolic Mint Extracts from Algeria on 7-Ketocholesterol-Treated Murine RAW 264.7 Macrophages
Source: Antioxidants (Basel). 2018 Dec 6;7(12):184. doi: 10.3390/antiox7120184 (PMC6315783; doi:10.3390/antiox7120184)
Supplement: Supplementary file 1 [file antioxidants-07-00184-s001.pdf]

Supplementary Table S1. Effect of mint extracts on cytokine secretion.

Samples	IL-6 (pg/mL)	IL-10 (pg/mL)	MCP-1 (pg/mL)	IFN-γ (pg/mL)	TNF-α (pg/mL)
**Ctl**	ND	ND	3,525.2 ± 176.3	ND	754.4 ± 37.8
**Vehicle**	ND	ND	4,345.8 ± 217.3	ND	879.5 ± 43.9
**LPS**	423.1 ± 21.2 *	156.7 ± 7.8 *	5,347.4 ± 267.5 *	ND	4,974.3 ± 248.5 *
**7KC20**	ND	ND	4,456.0 ± 222.8	ND	5,136.3 ± 256.8 #
**7KC20+LPS**	604.5 ± 30.3 $	ND	24,667.5 ± 1233.3 $	ND	15,162.5 ± 758.0 $
**MS200**	ND	45.5 ± 2.3 *	12,148.5 ± 607.3 *	ND	5,975.0 ± 298.8 *
**MS400**	ND	38.1 ± 1.9 *	10,939.8 ± 546.8 *	ND	7,828.5 ± 391.5 *
**MP200**	ND	ND	13,904.5 ± 4171.1 *	ND	8,645.0 ± 432.3 *
**MP400**	ND	31.6 ± 1.6 *	6,302.2 ± 315.0 *	ND	6,596.8 ± 329.8 *
**MR200**	ND	ND	5,395.3 ± 269.8 *	ND	2,519.3 ± 126.0 *
**MR400**	ND	ND	2,986.8 ± 149.3 *	ND	3,447.8 ± 172.3 *
**MS200+LPS**	90.0 ± 4.5 **	12.0 ± 0.6 **	4,106.3 ± 20.3 **	ND	4,579.0 ± 228.7
**MS400+LPS**	39.8 ± 2.0 **	78.0 ± 3.9 **	5,299.5 ± 264.7	ND	4467.7 ± 223.2
**MP200+LPS**	94.5 ± 4.8 **	46.3 ± 2.3 **	2,610.0 ± 130.5 **	ND	3,775.7 ± 188.7 **
**MP400+LPS**	39.0 ± 1.9 **	ND	2,988.7 ± 149.2 **	ND	2,745.0 ± 137.2 **
**MR200+LPS**	106.8 ± 5.2 **	10.3 ± 0.5 **	4,469.5 ± 223.5 **	ND	4,473.2 ± 223.5
**MR400+LPS**	50.8 ± 2.5 **	15.7 ± 0.8 **	7,824.0 ± 391	ND	2,955.2 ± 148.7 **
**MS200+7KC20**	6.3 ± 0.3 ##	ND	1,336.0±668.0	6.3±0.3 ##	14,198 ± 709.7 ##
**MS400+7KC20**	6.3 ± 0.3 ##	42.5 ± 2.1 ##	8,156.0±407.7	7.5±0.3 ##	11,096.0 ± 554.7 ##
**MP200+7KC20**	6.8 ± 0.4 ##	ND	10,567.2±528.2	ND	17,577.5 ± 878.7 ##
**MP400+7KC20**	6.2 ± 0.3 ##	ND	6,605.8±330.2	ND	17,985.5 ± 899.2 ##
**MR200+7KC20**	ND	ND	9,740.2±487.0	4.1±0.2 ##	10,479.0 ± 523.7 ##
**MR400+7KC20**	ND	ND	5,850.5±292.5	4.5±0.2 ##	10,340.2 ± 517.0.##
**MS200+7KC20+LPS**	1000.8 ± 50.0 $$	128.3 ± 6.5 $$	32,568.0 ± 1628.25	3.8 ± 0.1 $$	14,314.7 ± 715.5
**MS400+7KC20+LPS**	563.5 ± 28.0	56.2 ± 2.8 $$	15,428 ± 771.2	ND	6,816.5 ± 340.7
**MP200+7KC20+LPS**	653.8 ± 32.8	ND	22,541.0 ± 1127.0	4.0 ± 0.2 $$	968.5 ± 484.2
**MP400+7KC20+LPS**	287.5 ± 14.5 $$	ND	8,726.0 ± 436.2	ND	3,603.7 ± 180.0 $$
**MR200+7KC20+LPS**	463.5 ± 23.2 $$	ND	25,167.2 ± 1258.2	4.2 ± 0.2 $$	7,294.2 ± 364.5
**MR400+7KC20+LPS**	124.0 ± 6.3 $$	17.4 ± 0.9 $$	17,011.5 ± 850.5	3.5 ± 0.1 $$	5,551.5 ± 27.5

Data shown are mean ± SD from three independent experiments. They were analyzed by the ANOVA's test followed by a t test. A P value of 0.05 or less was considered as statistically significant ($p < 0.05$). No significant differences were observed between control (Ctl; untreated cells) and vehicle (ethanol 0.1%). *: comparison LPS, MS, MP and MR extracts versus Ctl; #: comparison 7KC20 versus vehicle; $: comparison (7KC20 + LPS) versus vehicle; **: comparison (MS, MP or MR extracts + LPS) versus LPS; ##: comparison (MS, MP or MR extracts + 7KC20) versus 7KC20; $$: comparison (MS, MP or MR extracts + 7KC20 + LPS) versus (7KC20 + LPS). Cytokine values lower than the limit of detection (IL-6: 5 pg/mL; IL-10: 17.5 pg/mL; MCP-1: 52.7 pg/mL; IFN-ϒ: 2.5 pg/mL; TNFα: 7.3 pg/mL) were noted as not detectable. ND: not detectable.

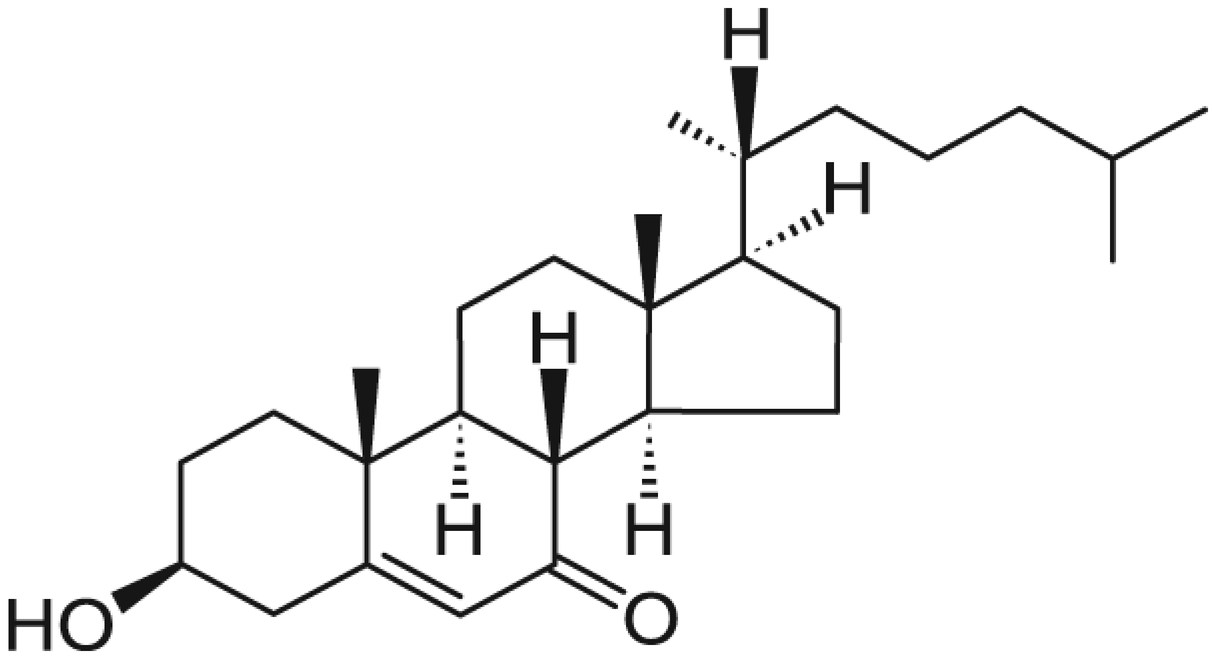

Supplementary Figure S1. 7-ketocholesterol (7KC) structure.
